# Delineating morbidity patterns in preterm infants at near-term age using a data-driven approach

**DOI:** 10.1186/s12887-024-04702-5

**Published:** 2024-04-11

**Authors:** Octavia-Andreea Ciora, Tanja Seegmüller, Johannes S. Fischer, Theresa Wirth, Friederike Häfner, Sophia Stoecklein, Andreas W. Flemmer, Kai Förster, Alida Kindt, Dirk Bassler, Christian F. Poets, Narges Ahmidi, Anne Hilgendorff

**Affiliations:** 1 Fraunhofer Institute for Cognitive Systems IKS, Munich, Germany; 2 Center for Comprehensive Developmental Care (CDeC(LMU)) at the Social Pediatric Center (iSPZ Hauner), LMU University Hospital, Ludwig-Maximilians-Universität München, Munich, Germany; 3 Institute for Lung Health and Immunity and Comprehensive Pneumology Center, Helmholtz Zentrum München, Member of the German Lung Research Center (DZL), Munich, Germany; 4 Department of Radiology, LMU University Hospital, Ludwig-Maximilians-Universität München, Member of the German Lung Research Center (DZL), Munich, Germany; 5 Division of Neonatology, Department of Pediatrics, Dr. von Hauner Children’s Hospital, LMU University Hospital, Ludwig-Maximilians-Universität München, Munich, Germany; 6 Metabolomics and Analytics Centre, LACDR, Leiden University, Leiden, Netherlands; 7 Department of Neonatology, University Hospital Zurich and University of Zurich, Zurich, Switzerland; 8 Department of Neonatology, University Children’s Hospital Tübingen, Tübingen, Germany

**Keywords:** Preterm birth, Morbidity co-occurrence, Bronchopulmonary dysplasia, Risk profile correlation, Morbidity correlation, Clustering, Machine learning

## Abstract

**Background:**

Long-term survival after premature birth is significantly determined by development of morbidities, primarily affecting the cardio-respiratory or central nervous system. Existing studies are limited to pairwise morbidity associations, thereby lacking a holistic understanding of morbidity co-occurrence and respective risk profiles.

**Methods:**

Our study, for the first time, aimed at delineating and characterizing morbidity profiles at near-term age and investigated the most prevalent morbidities in preterm infants: bronchopulmonary dysplasia (BPD), pulmonary hypertension (PH), mild cardiac defects, perinatal brain pathology and retinopathy of prematurity (ROP). For analysis, we employed two independent, prospective cohorts, comprising a total of 530 very preterm infants: AIRR (“Attention to Infants at Respiratory Risks”) and NEuroSIS (“Neonatal European Study of Inhaled Steroids”). Using a data-driven strategy, we successfully characterized morbidity profiles of preterm infants in a stepwise approach and (1) quantified pairwise morbidity correlations, (2) assessed the discriminatory power of BPD (complemented by imaging-based structural and functional lung phenotyping) in relation to these morbidities, (3) investigated collective co-occurrence patterns, and (4) identified infant subgroups who share similar morbidity profiles using machine learning techniques.

**Results:**

First, we showed that, in line with pathophysiologic understanding, BPD and ROP have the highest pairwise correlation, followed by BPD and PH as well as BPD and mild cardiac defects. Second, we revealed that BPD exhibits only limited capacity in discriminating morbidity occurrence, despite its prevalence and clinical indication as a driver of comorbidities. Further, we demonstrated that structural and functional lung phenotyping did not exhibit higher association with morbidity severity than BPD. Lastly, we identified patient clusters that share similar morbidity patterns using machine learning in AIRR (n=6 clusters) and NEuroSIS (n=8 clusters).

**Conclusions:**

By capturing correlations as well as more complex morbidity relations, we provided a comprehensive characterization of morbidity profiles at discharge, linked to shared disease pathophysiology. Future studies could benefit from identifying risk profiles to thereby develop personalized monitoring strategies.

**Trial registration:**

AIRR: DRKS.de, DRKS00004600, 28/01/2013. NEuroSIS: ClinicalTrials.gov, NCT01035190, 18/12/2009.

**Supplementary Information:**

The online version contains supplementary material available at 10.1186/s12887-024-04702-5.

## Background

### Preterm birth - a relevant challenge for health worldwide

More than 10% of infants worldwide are born preterm [1, 2] and the resulting morbidities rank amongst the top ten causes for impaired health worldwide [3, 4]. With the improvement in neonatal intensive care, survival rates have continuously increased [5], accompanied by growing incidences of long-term complications. Long-term impairment results from the most prevalent morbidities diagnosed at near-term age, affecting several organs, systems and functions, dominated by the cardio-respiratory system and the central nervous system [1, 6, 7]. Morbidity prevalence is determined by the degree of immaturity as well as the impact of different pre- and postnatal insults [8] including necessary treatments such as mechanical ventilation or oxygen supplementation and the inevitable exposure of an immature immune system to viruses and bacteria.

### Morbidity burden as a consequence of prematurity

One of the most prevalent morbidities with significant long-term implications in preterm infants is chronic lung disease, i.e., bronchopulmonary dysplasia (BPD) [9, 10]. Defined by the expert consensus conference in 2001 [11], BPD is divided into three severity grades and results from the exposure of the structurally and functionally immature lung to pre- and postnatal hazards. The provoked micro-injuries as well as the exhaustion of "defense" and repair strategies, e.g., through exposure to oxygen radicals, trigger inflammatory processes and impact lung maturation, resulting in alveolar hypoplasia, interstitial remodeling and injury to the pulmonary vascular bed.

Morbidities associated with prematurity rarely occur isolated [8] and BPD is considered a critical confounder for health development in preterm infants. For instance, BPD is associated with higher prevalence of patent ductus arteriosus (PDA) [12], atrial septal defect (ASD), and pulmonary hypertension (PH) [13–16]. It affects the neurosensory development [17, 18] and is related to brain lesions like intraventricular hemorrhage (IVH) or white matter injury of prematurity (WMI) [19] as well as an increased risk for retinopathy of prematurity (ROP) [20–22].

The expert community has discussed a variety of reasons for morbidity co-occurrence. These include the incidence of hypoxic or hypercapnic episodes in BPD infants [21], shared pathophysiologic processes triggered through comparable insults [23] as well as lung-derived mitigation of inflammatory signals contributing to extra-pulmonary manifestation of disease [21, 24].

### The need for a holistic view on the morbidity landscape

Complications of the cardio-respiratory and central nervous systems are the most prevalent conditions after premature birth and their occurrence leads to significant short, middle and long-term consequences in preterm born infants, children and adults. Since these morbidities often manifest together, their co-occurrence may lead to complex interdependencies and associated clinical patterns determining the infant’s health development. A comprehensive understanding of the morbidity landscape at near-term age could help clinicians to define risk profiles that could inform follow-up strategies.

Although the co-occurrence of neurosensory and cardiovascular pathologies [20] as well as individual morbidity associations have already been investigated [25], two essential challenges remain: (1) most studies have not looked beyond pairwise morbidity associations and therefore do not fully capture the complex morbidity landscape including more complex co-occurrence patterns, and (2) variations in cohorts related to study design, inclusion/exclusion criteria as well as statistical methods and result reporting across studies most often hinder comparability between findings. As a result, the correlation strength between a morbidity pair reported in one study cannot be easily compared with the correlation strength of a different morbidity pair reported in another study, making it difficult to develop a broader clinical view on morbidity profiles at near-term age, i.e., hospital discharge.

### Research aims of our study

In this study, we investigate morbidity interdependencies of the spectrum outlined above thereby addressing the limitations in this field in order to provide a comprehensive understanding of morbidity profiles in very preterm infants at near-term age. By moving beyond individual correlations and analyzing morbidities collectively, we aim to identify frequent co-occurrence patterns and patient subgroups with particular morbidity profiles using a stepwise data-driven approach. We define these profiles based on the most prevalent morbidities of the cardio-respiratory and central nervous systems, assessed upon discharge from neonatal care according to international criteria. Investigating the morbidity patterns in relation to BPD - although criticized for its lack in discriminatory and predictive power [13, 24] - allows us to study a potential driver for morbidity development [9, 21]. At the same time, we took the opportunity to assess the potential of deeper phenotyping strategies for the diagnostic performance as an indicator of morbidity risk.

Our main contributions are as follows: (1) comprehensive analysis of morbidity correlations and profiles at near-term age, (2) assessment of the capacity of BPD as well as associated structural and functional lung abnormalities to characterize the severity of morbidities, (3) identification of patient subgroups and potential risk profiles at near-term age and (4) assessment of the generalizability of our findings by leveraging two independent cohorts.

## Methods

### Cohorts

To increase robustness of our analysis, we leveraged data from a German and a European cohort of very preterm infants ($<32$ and $<28$ weeks gestational age (GA), respectively). Both datasets focused on BPD, while carefully characterizing all infants according to international criteria for morbidity development. Our “Attention to Infants at Respiratory Risks” (AIRR) cohort, from Ludwig Maximilian University, served as the primary cohort for approach development, while result validation was performed on data derived from our phase-III clinical trial “The Neonatal European Study of Inhaled Steroids” (NEuroSIS) from University Hospital Tübingen.

#### Primary cohort: AIRR

The AIRR cohort prospectively included preterm infants born at < 32 weeks GA at the Perinatal Center of Ludwig Maximilian University, Munich (Campus Grosshadern) between 01/2012 and 12/2020 after informed parental consent (Ethics Committee LMU #195-07; German Registry for Clinical Studies DRKS #00004600). In n=176 infants, the pre-, peri- and postnatal clinical course was comprehensively monitored including prevalent morbidities of the cardio-respiratory and central nervous systems (Additional file 1, Supplementary material 1). One infant died during neonatal intensive care before discharge. At around 36 weeks postmenstrual age (PMA), study infants underwent brain and lung magnetic resonance imaging (MRI) upon informed consent.

#### Validation cohort: NEuroSIS

The NEuroSIS study (phase-III clinical trial, randomized controlled trial (RCT)) prospectively included 863 infants < 28 weeks GA born between 04/2010 and 08/2013 with the need for positive-pressure ventilation (Ethics Committee Tübingen #112/2022A; ClinicalTrials.gov number, NCT01035190) [26]. As infants of the intervention group received budesonide until no longer requiring oxygen and positive-pressure support or until reaching 32+0 weeks PMA, only infants of the control group having received placebo (hydrofluoroalkane propellant) treatment were considered for analysis (n=422 patients). In these infants, the pre-, peri- and postnatal clinical course was comprehensively monitored including prevalent morbidities of the cardio-respiratory and central nervous systems (Additional file 1, Supplementary material 2). 57 of the placebo treated patients died during neonatal intensive care before discharge. Regulatory approvals and written informed parental consent were obtained before study randomization.

### Definitions of morbidities at near-term age

We focused on the most prevalent morbidities defined by international criteria to characterize the morbidity profile of preterm infants at near-term age, i.e., at discharge from neonatal care (AIRR: $38.05 \pm 2.78$ weeks PMA; NEuroSIS: $40.30 \pm 4.57$ weeks PMA). They capture conditions of the cardio-respiratory as well as the central nervous systems.

**Bronchopulmonary dysplasia (BPD)** was defined according to the NICHD/NHLBI/ORD consensus [11] with disease grading assigning infants to three groups: mild BPD for oxygen (O_2_) supplementation for 28 days and no need for O_2_ supplementation at 36 weeks PMA; moderate BPD for fraction of inspired oxygen (FiO_2_) $< 0$.30 at 36 weeks PMA; severe BPD for FiO_2_ $\ge 0.30$ at 36 weeks PMA and/or positive pressure (treatment defined as continuous application, one day of treatment = O_2_ supplementation $>12$ hours).

In both cohorts, echocardiography was performed upon clinical indication and included the evaluation of indicators for pulmonary hypertension (PH) or post tricuspid shunts comprising intracardiac (e.g., ASD) or extracardiac (e.g., PDA) shunting with or without hemodynamic relevance [27].

**Pulmonary Hypertension (PH)** was diagnosed by echocardiography according to international criteria as right heart enlargement, pulmonary artery enlargement and tricuspid valve insufficiency in the presence of clinical abnormalities [28, 29].

**Mild cardiac defects** were diagnosed by echocardiography and included the presence of post-tricuspid shunting, i.e., PDA and/or ASD, as indicators of and risk factors for adverse pulmonary circulation. The presence of any of both pathologies is represented as a binary morbidity. Hemodynamically relevant shunting through the PDA was either treated with Cyclooxygenase-2 inhibitors or surgical ligation was performed in case of unresponsiveness to drug treatment.

**Perinatal brain pathology** was detected by cranial ultrasound (both cohorts) and/or brain MRI (AIRR cohort) [30], and assessed by an experienced neonatologist or radiologist, respectively. We distinguished three levels of perinatal brain pathology: none, mild, and severe. No brain pathology was defined as the absence of IVH [31] or WMI [32] in cranial ultrasound (NEuroSIS and AIRR) and/or other brain pathology (MRI, AIRR cohort). Mild brain pathology was defined as IVH grade 1 or 2 (crainal ultrasound, NEuroSIS and AIRR) and/or the presence of septum pellucidum agenesis, age-inappropriate myelination, mild asymmetry of the lateral ventricles or status after meningoencephalitis (MRI, AIRR cohort). Severe brain pathology included all cases with IVH grades 3 and 4 and/or WMI (carinal ultrasound, NEuroSIS and AIRR) and/or additional pathology such as pathologic arterial flow profile, cortical edema or -necrosis, sinus vein thrombosis or candida abscesses in MRI (AIRR cohort).

**Retinopathy of prematurity (ROP)** was assessed by fundoscopy through an experienced ophthalmologist and classified according to international criteria [33] distinguishing five disease grades (grade 1: demarcation line between normal and immature retina; grade 2: raised border ridge between normal and immature retina; grade 3: extraretinal neovascular proliferation or flat neovascularization; grade 4: partial retinal detachment; grade 5: complete retinal detachment).

### Structural and functional lung phenotyping by MRI

In the AIRR cohort, preterm infants underwent 3-Tesla MRI (Magnetom Skyra, Siemens Healthineers, Erlangen, Germany) around the time of BPD diagnosis (i.e., 36 weeks PMA) in unsedated, spontaneous sleep breathing room air (FiO_2_ = 0.21). The infants were laying in a vacuum mattress and wore neonatal noise attenuators (MiniMuffs®, Natus® newborn care, Seattle, USA) for hearing protection.

**Structural lung abnormalities** Structural lung abnormalities were scored in T2-weighted single-shot fast-spin-echo sequences in coronal, axial and sagittal orientations of n=70 infants included in the present analysis using a five-point Likert scale addressing interstitial and airway remodeling, emphysematous changes and ventilation inhomogeneity at the time of the BPD diagnosis (mean age at MRI 36.94 ± 3.88 weeks) and summarized as a total score as published previously by Förster et al. [34], where low values indicated less structural lung abnormalities.

**Pulmonary artery (PA) flow** Variables of PA flow, i.e. net forward volume (NFV) measured in both PAs (ratio right-over-left) was quantified as published by Häfner et al. [35] using HASTE acquisitions, T1-weighted images, T1 and T2 mapping sequences, cardiac cine sequences, and phase-contrast flow measurements of n=85 infants included in the present analysis at the time of BPD diagnosis (mean age at MRI 36.0 ± 3.54 weeks).

### Perinatal variables

The AIRR study comprehensively monitored the pre-, peri- and postnatal course until discharge including more than 300 variables of cardio-respiratory, neurodevelopmental, metabolic and general health (Additional file 1, Supplementary material 1). The NEuroSIS study monitored the peri- and postnatal course of the preterm infants until discharge including more than 200 variables collected during hospital stay [26] (Additional file 1, Supplementary material 2).

### Statistical analyses

From n=176 patients in the AIRR cohort, n=5 patients had to be excluded, resulting in a total of n=171 patients for final analysis. One patient had to be excluded due to death before discharge, two patients were excluded due to GA $> 32$ weeks, and two patients were excluded due to value missingness for at least one of the morbidities. From n=422 patients in the NEuroSIS control group, n=63 patients had to be excluded, resulting in a total of n=359 patients for final analysis. 57 patients had to be excluded due to death before discharge and six patients were excluded due to value missingness for at least one of the morbidities. In NEuroSIS, data for MRI assessment of perinatal brain pathology was not available. The list of variables used in our analysis is shown in Additional file 1, Supplementary Table 1.

To assess differences between cohorts, we performed a comparative analysis of patients’ characteristics based on observed counts, percentages and the chi-squared test for qualitative variables and mean, standard deviation and the Mann-Whitney U test for quantitative variables, accounting for multiple testing using Bonferroni correction. We employed a stepwise, data-driven approach to provide a comprehensive overview of the morbidity profiles in very preterm infants at near-term age, comprising the following analysis steps:

1. **Pairwise correlations:** To measure the strength of monotonous association between all morbidities pairwise, we computed Kendall’s $\tau$ correlation coefficient per morbidity pair. We used Bonferroni correction to account for multiple testing.
2. **Discriminatory power of BPD:** We provided a more detailed analysis of BPD, assessing its capacity to characterize the morbidity severity. We investigated the differences between BPD groups (according to the clinical definition) with respect to morbidity incidences, relative risks (RRs) and odds ratios (ORs). Here, *risk* describes the probability of patients exhibiting a particular morbidity within a designated group (i.e., a specific BPD grade) relative to the reference group (i.e., no BPD). An additional analysis step was performed on the AIRR cohort only to evaluate whether structural (n=70 patients) and functional (n=85 patients) lung phenotyping by MRI could improve correlation with discharge morbidities compared to the clinical BPD diagnosis.
3. **Co-occurrence patterns:** We went beyond pairwise correlations and explored collective co-occurrence of morbidities to capture morbidity profiles and their frequency. Throughout this study, we use the term *morbidity load* to denote the number of morbidities observed in a profile, with no morbidity load describing the absence of all morbidities, and high morbidity load describing the presence of at least four out of five morbidities.
4. **Clustering of morbidity profiles:** We employed agglomerative hierarchical clustering with complete linkage, an unsupervised machine learning technique, to identify patient clusters based on similar morbidity profiles at discharge. To treat all morbidities with equal importance for clustering, we used normalized ranks to scale disease severity for BPD, perinatal brain pathology, and ROP, thereby displaying all morbidities with values between 0 and 1. For measuring (dis-)similarity between patients, we computed the Gower’s distance of all patients pairs, with 0 indicating identical morbidity profiles and 1 indicating maximal dissimilar morbidity profiles. The Gower’s distance is suitable for mixed type data, i.e., variables which are binary (i.e., PH and mild cardiac defects) and ordinal (i.e., BPD, perinatal brain pathology, and ROP). To identify the optimal number of clusters k, we repeated the clustering with varying k and compared the silhouette coefficients. Based on their similarities, patients were assigned to one of the clusters, resulting in groups of patients whose morbidity patterns are more similar to each other than to the rest of the patients. To better understand each cluster, we characterized them by the distribution of morbidities and postnatal variables.

All analyses were performed using Python version 3.8.10. The statistical testing was performed using the package scipy (version 1.10.0) [36] and the clustering analysis was performed using scikit-learn (version 1.0.1) [37].

## Results

### Cohort characteristics

The two cohorts AIRR and NEuroSIS revealed statistically significant differences in gestational age, morbidity incidences (and severity) of PH, mild cardiac defects, perinatal brain pathology and ROP, with only BPD rates showing no significant difference between cohorts (Table 1).

**Table 1:** Patient characteristics in the AIRR and NEuroSIS cohorts including statistical comparisons

	AIRR	NEuroSIS
	*n* = 171	*n* = 359
**GA [weeks]** ^1,∗^	27.14 ± 2.19	26.23 ± 1.11
**BW [g]** ^1^	918.65 ± 311.29	821.69 ± 177.31
**Sex** (male)^2^	90 (52.63%)	180 (50.14%)
**Multiple birth** (yes)^2^	57 (33.33%)	75 (20.89%)
**Umbilical artery pH** ^1,∗^	7.34 ± 0.08	7.30 ± 0.11
**1-minute Apgar** ^1,∗^	5.92 ± 1.84	4.99 ± 2.31
**5-minute Apgar** ^1,∗^	7.91 ± 1.16	6.96 ± 1.73
**ANCS** ^2,∗^	135 (81.82%)	333 (92.76%)
**Hospital stay [d]** ^1,∗^	77.19 ± 27.13	98.80 ± 34.84
**EOI** (yes)^2^	33 (19.76%)	106 (29.53%)
**BPD** ^1^		
None	59 (34.50%)	115 (32.03%)
Mild	58 (33.92%)	94 (26.18%)
Moderate	24 (14.04%)	39 (10.86%)
Severe	30 (17.54%)	111 (30.92%)
**PH** (yes)^2,∗^	15 (8.77%)	4 (1.11%)
**Mild cardiac defect** (yes)^2,∗^	58 (33.92%)	190 (52.92%)
**Perinatal brain pathology** ^1,∗^		
None	107 (62.57%)	154 (42.90%)
Mild	44 (25.73%)	170 (47.35%)
Severe	20 (11.70%)	35 (9.75%)
**ROP** ^1,∗^		
None	122 (71.35%)	186 (51.81%)
Grades 1/2	38 (22.22%)	127 (35.38%)
Grades$\ge$3	11 (6.43%)	46 (12.81%)

For quantitative variables, we indicate the mean ± standard deviation, while for qualitative variables, we indicate the count and percentage (relative to the total number of patients having that particular variable observed)

*Abbreviations*: *GA* Gestational age, *BW* Birth weight, *ANCS* Antenatal corticosteroids, *EOI* Early onset infection [38], *BPD* Bronchopulmonary dysplasia [11], *PH* Pulmonary hypertension [28, 29], *ROP* Retinopathy of prematurity [33]

^*^ Statistically significant difference between cohorts after Bonferroni correction (adjusted *p*-value < 0.05)

^1 ^The differences between cohort was assessed using Mann-Whitney U test

^2^ The differences between cohort was assessed using chi-squared test

### Pairwise correlations between morbidities

By computing pairwise correlations between all morbidity pairs, we quantitatively measured the strength of association between individual morbidities, while ensuring comparability between coefficients within cohorts. While all observed correlation coefficients are $\le 0.3$, we described the observed significant correlations as indicators of morbidity association, considering the complex nature of the morbidities included and their interdependencies.

In both cohorts, two out of ten possible morbidity pairs showed statistically significant correlations. Consistent in both cohorts, BPD and ROP exhibited the highest correlation (AIRR: $\tau$=0.306, NEuroSIS: $\tau$=0.194). While in AIRR, BPD was significantly correlated with PH ($\tau$=0.212), this was not replicated in NEuroSIS. Noteworthy is also the statistically significant correlation between BPD and mild cardiac defects in NEuroSIS ($\tau$=0.185). No significant correlation was observed between BPD and perinatal brain pathology in either of the cohorts. All correlations are summarized in Table 2.

**Table 2:** Pairwise correlation between morbidities

	1	2	3	4	5
(a) AIRR					
1 BPD	-	0.212*	0.148	0.034	0.306*
2 PH	0.212*	-	0.040	0.038	0.122
3 Mild cardiac defect	0.148	0.040	-	-0.128	0.122
4 Perinatal brain pathology	0.034	0.038	-0.128	-	-0.041
5 ROP	0.306*	0.122	0.122	-0.041	-
(b) NEuroSIS					
1 BPD	-	0.087	0.185*	0.065	0.194*
2 PH	0.087	-	0.100	0.077	0.007
3 Mild cardiac defect	0.185*	0.100	-	0.041	0.110
4 Perinatal brain pathology	0.065	0.077	0.041	-	0.043
5 ROP	0.194*	0.007	0.110	0.043	-

Entries represent Kendall’s $\tau$ coefficient measuring strength of correlation. The morbidities BPD, perinatal brain pathology, and ROP are ordinal, whereas PH and mild cardiac defects are binary

^*^Statistically significant correlation after Bonferroni correction (adjusted *p*-value < 0.05)

### Discriminatory power of BPD among morbidity occurrence

As BPD serves as a key indicator of lung development and is interconnected with other morbidities, we assessed the capacity of BPD to delineate the morbidity patterns at near-term age in preterm infants in both cohorts. We quantified morbidity occurrence, incidence, RR, and OR for all BPD severity groups, and thereby described the relationship between BPD grades and morbidities as well as estimated the increase of morbidity risk per BPD group relative to the group without BPD. In our analyses, we relied on RR (Table 3) as a measure for risk increase, since OR tends to overestimate the effect size. However, for a comprehensive overview, we reported RR and OR to allow for comparisons with previous studies (Additional file 1, Supplementary Table 2). In the AIRR cohort, the risk of developing any morbidity for patients with mild BPD showed no statistically significant increase compared to patients without BPD. For cases with moderate BPD, a statistically significant increased risks for PH (RR=12.29) and ROP grades 1/2 (RR=2.81) were observed. In severe BPD cases, we observed a ninefold increase in risk for PH and severe ROP (RR=9.83) as well as a threefold increase in ROP grades 1/2 (RR=3.65). Mild cardiac defects or perinatal brain pathology showed no significant differences between BPD severity groups (Table 3a).

In NEuroSIS, the highest increase in risk for infants with BPD was observed for severe ROP, showing a sixfold increase in mild (RR=6.73) and an over elevenfold increase in moderate (RR=11.79) and severe (RR=12.95) BPD cases. The risk for mild cardiac defects was 60% higher in cases with moderate and severe BPD when compared to patients without the disease. Comparable to the results obtained in the AIRR cohort, none of the BPD severity groups showed a significantly increased risk for perinatal brain pathology. PH could not be assessed in this context due to limited incidence numbers (Table 3b).

**Table 3:** Morbidity prevalences and RRs stratified by BPD severity groups

(a) AIRR				
	**No BPD**	**Mild BPD**	**Moderate BPD**	**Severe BPD**
	n = 59	n = 58	n = 24	n = 30
**PH**				
Count	1 (1.69%)	4 (6.9%)	5 (20.83%)	5 (16.67%)
RR	-	4.07 (0.47-35.32)	**12.29 (1.51-99.77)**	** 9.83 (1.20-80.43)**
**Mild cardiac defect**				
Count	16 (27.12%)	17 (29.31%)	11 (45.83%)	14 (46.67%)
RR	-	1.08 (0.61-1.93)	1.69 (0.92-3.09)	1.72 (0.98-3.03)
**Perinatal brain pathology (mild)**				
Count	10 (16.95%)	18 (31.03%)	7 (29.17%)	9 (30.0%)
RR	-	1.83 (0.93-3.62)	1.72 (0.74-3.99)	1.77 (0.81-3.88)
**Perinatal brain pathology (severe)**				
Count	8 (13.56%)	8 (13.79%)	1 (4.17%)	3 (10.0%)
RR	-	1.02 (0.41-2.53)	0.31 (0.04-2.33)	0.74 (0.21-2.58)
**ROP (grades 1/2)**				
Count	7 (11.86%)	10 (17.24%)	8 (33.33%)	13 (43.33%)
RR	-	1.45 (0.59-3.56)	**2.81 (1.15-6.89)**	**3.65 (1.63-8.18)**
**ROP (grades** $\ge$ **3)**				
Count	1 (1.69%)	4 (6.9%)	1 (4.17%)	5 (16.67%)
RR	-	4.07 (0.47-35.32)	2.46 (0.16-37.73)	**9.83 (1.20-80.43)**
(b) NEuroSIS				
	**No BPD**	**Mild BPD**	**Moderate BPD**	**Severe BPD**
	n = 115	n = 94	n = 39	n = 111
**PH**				
Count	0 (0.0%)	1 (1.06%)	0 (0.0%)	3 (2.7%)
RR	-	- (-)	- (-)	- (-)
**Mild cardiac defect**				
Count	46 (40.0%)	48 (51.06%)	25 (64.1%)	71 (63.96%)
RR	-	1.28 (0.95-1.72)	**1.6 (1.16-2.22)**	** 1.6 (1.23-2.08)**
**Perinatal brain pathology (mild)**				
Count	50 (43.48%)	42 (44.68%)	18 (46.15%)	60 (54.05%)
RR	-	1.03 (0.76-1.40)	1.06 (0.71-1.58)	1.24 (0.95-1.63)
**Perinatal brain pathology (severe)**				
Count	10 (8.7%)	10 (10.64%)	6 (15.38%)	9 (8.11%)
RR	-	1.22 (0.53-2.81)	1.77 (0.69-4.55)	0.93 (0.39-2.21)
**ROP (grades 1/2)**				
Count	38 (33.04%)	38 (40.43%)	16 (41.03%)	35 (31.53%)
RR	-	1.22 (0.86-1.75)	1.24 (0.79-1.96)	0.95 (0.65-1.39)
**ROP (grades** $\ge$ **3)**				
Count	2 (1.74%)	11 (11.7%)	8 (20.51%)	25 (22.52%)
RR	-	** 6.73 (1.53-29.61)**	**11.79 (2.62-53.20)**	** 12.95 (3.14-53.39**)

Displayed for each BPD severity group are: morbidity count with the corresponding incidence relative to the size of the respective BPD severity group and the relative risk (RR) with the corresponding 95% confidence interval (CI). Statistically significant (i.e., CI does not include 1) RR values are indicated in bold

### Structural and functional lung phenotyping does not improve correlation with discharge morbidities

Given the limited discriminatory power observed for the clinical BPD diagnosis within the morbidity patterns, we evaluated whether additional imaging-derived variables on structural and functional changes to the lung could exhibit stronger correlations with the morbidities analyzed than the clinical diagnosis of BPD. We computed the correlations for all morbidities in the two subsets of patients for which these additional variables were available. Structural lung abnormalities, assessed in MRI by consensus scoring (n=70 patients), and PA flow (n=85 patients) showed only weak non-significant correlations with the other morbidities, therefore not improving the overall discriminatory power within morbidity patterns (Additional file 1, Supplementary Table 3).

### Morbidity co-occurrence and morbidity patterns

**Fig. 1:**
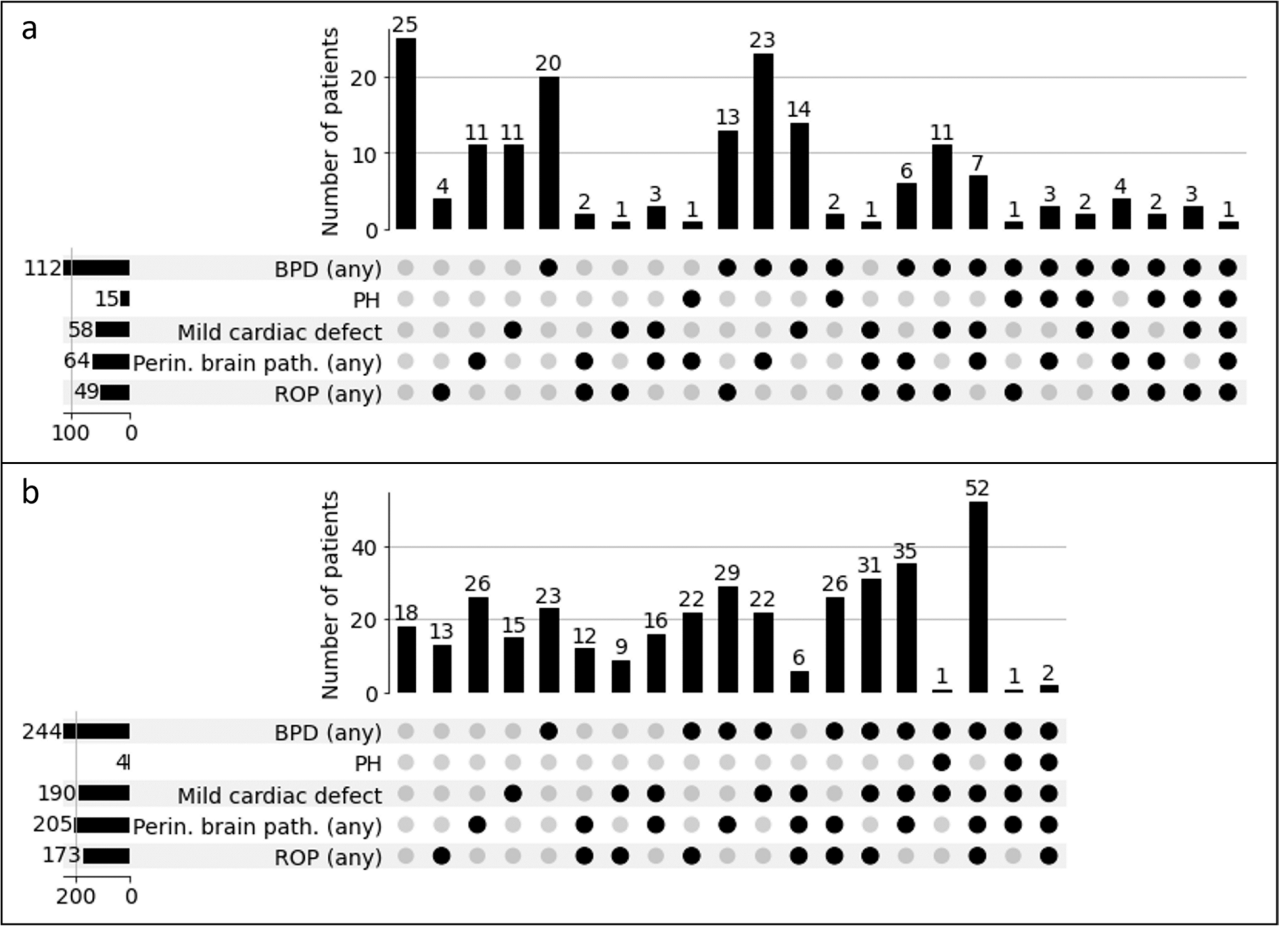
Morbidity pattern distribution in (**a**) AIRR and (**b**) NEuroSIS. Each column corresponds to a different morbidity pattern, with the black dots indicating the morbidities present in each pattern and the vertical bar showing the number of patients exhibiting the respective pattern. The horizontal bars on the left indicate the total number of patients exhibiting each morbidity. For visualization purposes, we used a binary representation of morbidities, displaying a black dot for any severity of BPD, perinatal brain pathology, and ROP

Since individual correlations might not fully capture complex patterns of morbidity co-occurrence, we next considered all morbidities simultaneously for analysis. For visualization of morbidity profile distribution, we used a binary representation of morbidities (i.e., for BPD, brain pathology, and ROP, morbidity is shown for any severity grade in Fig. 1 and for moderate to severe grades only in Additional file 1, Supplementary Fig. 1).

In AIRR, we observed a total of 24 unique patterns (Fig. 1a). The most frequent pattern in AIRR was no morbidity load (n=25, 14.6%), i.e., absence of BPD, PH, mild cardiac defects, perinatal brain pathology and ROP. A total of n=46 (26.9%) patients were characterized by the occurrence of one morbidity only, i.e., perinatal brain pathology (n=11, 6.4%), mild cardiac defects (n=11, 6.4%), any BPD grade (n=20, 11.7%) and any ROP grade (n=4, 2.3%). PH was not observed as a single morbidity. The presence of two morbidities was observed in n=59 (34.5%) patients, whereas three morbidities were observed in n=31 (18.1%) patients. Four to five morbidities, i.e., a high morbidity load, were observed in n=9 (5.3%) and n=1 (0.6%) patients, respectively.

In NEuroSIS, we observed 19 combinations of morbidity co-occurrence (Fig. 1b). The most prevalent pattern in NEuroSIS included n=52 (14.5%) patients characterized by BPD, mild cardiac defects, perinatal brain pathology, and ROP. No morbidity load (i.e., absence of all morbidities) was observed in n=18 (5.0%) patients. Single morbidity patterns occurred in n=77 (21.4%) patients, where n=26 (7.2%) suffered from perinatal brain pathology, n=15 (4.2%) from mild cardiac defects, n=23 (6.4%) from BPD, and n=13 (3.6%) from ROP. Two and three morbidities were observed in n=110 (30.6%) and n=99 (27.6%) patients, respectively. A high morbidity load was observed in n=53 (14.8%) and n=2 (0.6%) patients, with four and five morbidities, respectively.

### Identification of patient clusters sharing similar morbidity profiles

To identify patient clusters based on similar morbidity profiles, we performed identical clustering analysis in both cohorts independently using the five morbidities as an input. Computing silhouette coefficients for different numbers of clusters revealed *six* as the most suitable number of clusters in AIRR and *eight* in NEuroSIS (Additional file 1, Supplementary Fig. 2). The resulting cluster sizes are depicted in Additional file 1, Supplementary Fig. 3.

**Fig. 2:**
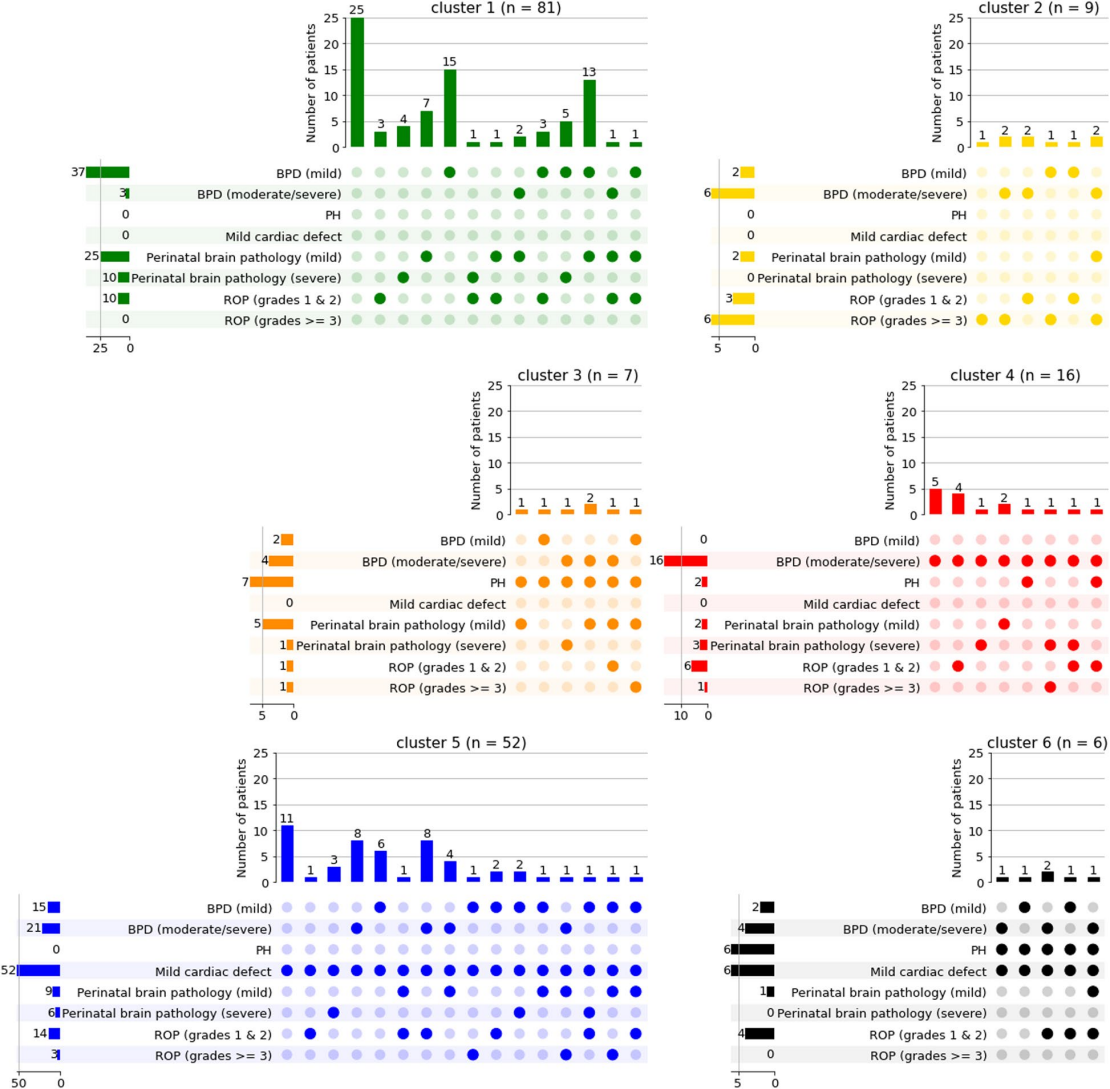
Distribution of morbidity patterns per clusters in AIRR cohort. The binarization of BPD and ROP grades was done for visualization purposes, while the clustering analysis considered actual BPD and ROP grades as input

**Fig. 3:**
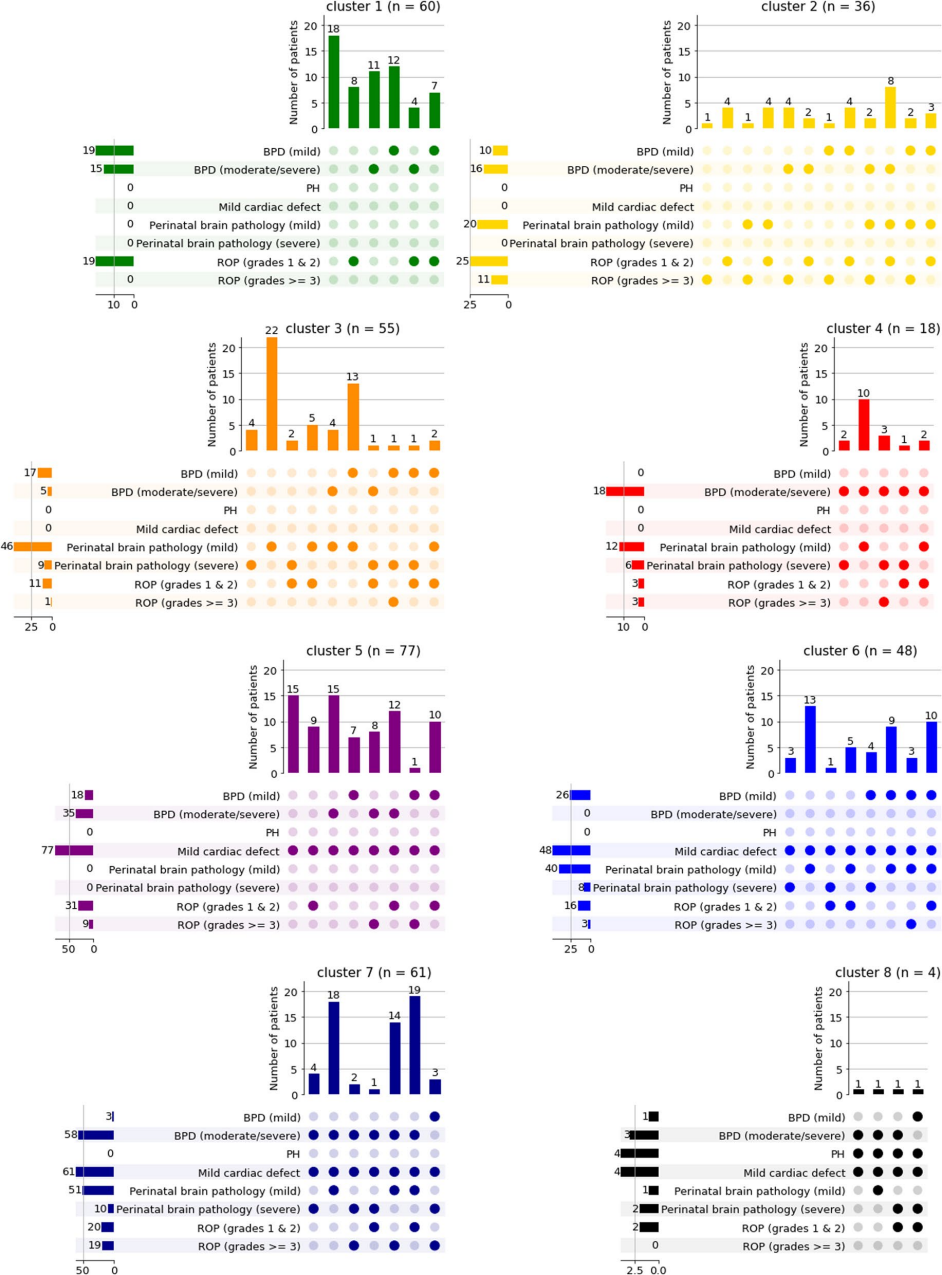
Distribution of morbidity patterns per clusters in NEuroSIS cohort. The binarization of BPD and ROP grades was done for visualization purposes, while the clustering analysis considered actual BPD and ROP grades as input

For visualization, we ordered the clusters of patients based on morbidity load. While the identified clusters overall differed between AIRR (Fig. 2) and NEuroSIS (Fig. 3), important similarities could be demonstrated. In both cohorts, cluster 1 (green) encompassed no or a low morbidity load, none of whom included PH, mild cardiac defects, or severe ROP. Perinatal brain pathology was only observed in some patients (AIRR cohort). Cluster 2 (yellow) was described by the absence of PH, mild cardiac defects, and severe perinatal brain pathology in both cohorts as well as the presence of mild perinatal brain pathology, BPD and ROP in differing severities. Cluster 3 (orange) was characterized by the absence of mild cardiac defects in both cohorts, in addition to the presence of PH in AIRR. Cluster 4 (red) showed moderate to severe BPD in all patients in the absence of mild cardiac defects. Clusters 5 and 6 (AIRR) as well as 5 to 8 (NEuroSIS) were characterized by the presence of mild cardiac defects in all patients and showed an overall increase in morbidity load. All patients in cluster 6 (AIRR) and cluster 8 (NEuroSIS) were diagnosed with PH.

Prevalence of morbidities per cluster are shown in Additional file 1, Supplementary Fig. 4. A detailed characterization of the patients in each cluster was enabled by integration of perinatal variables (Additional file 1, Supplementary Fig. 5), demonstrating an even distribution without notable differences in perinatal variable prevalence among clusters.

## Discussion

Morbidities determine life expectancy as well as long-term quality of life in the ever-growing population of preterm infants. Different studies associated co-occurring morbidities and specifically outlined the potential of BPD as a driver of cardiac and central nervous system complications [17, 39]. Significantly adding to previous observations that focused mostly on individual morbidities and analyzed single correlations, we, for the first time, employed a holistic data-driven approach to provide a comprehensive understanding of morbidity profiles upon discharge, i.e., at near-term age, in two prospective preterm cohorts. Conducting our analyses in two cohorts with differing demographic profiles highlights generalizability of the findings obtained on the one hand, and points to differences stemming from the degree of clinical phenotyping on the other. We systematically quantified all pairwise correlations between the selected morbidities, demonstrating BPD as the only morbidity displaying significant correlations with other morbidities in both cohorts. Considering BPD as a potential driver of comorbidity development as indicated by clinical data, we next addressed the differences in morbidity risk between BPD severity groups, revealing the limited capacity of BPD to delineate morbidity patterns at discharge. Likewise, neither structural nor functional lung phenotyping by MRI improved the correlation with discharge morbidities in comparison to the clinical BPD diagnosis. This finding likely points to the need for structural or functional markers that better relate to shared pathophysiologic or risk factor pattern when addressing morbidity co-occurrence. To complement the pairwise analysis of morbidities, we investigated co-occurrences of all morbidities simultaneously, capturing morbidity profiles as a whole. In a final step, hierarchical clustering facilitated the identification of patients clusters by morbidity profile similarity.

### Unraveling morbidity relations at neonatal intensive care unit (NICU) discharge under specific consideration of BPD

When investigating morbidity pairs, the only significant correlations engaged BPD. While within the range of weak correlation, they provide indication of association between BPD and ROP (Table 2), further supported when analyzing BPD severity groups (Table 3). The significant increase in risk for developing severe ROP in the presence of severe (AIRR, NEuroSIS) or even mild or moderate BPD (NEuroSIS) can be explained by shared pathophysiologic hallmarks between both diseases with dysfunctional angiogenic signaling and vessel formation [40, 41], presence of shared inflammatory processes and genetic risk factors [42]. The interrelation highlights the need to consider both complications when designing future treatment and follow-up strategies [20–22]. Likewise, the correlation of BPD and PH together with their co-occurrence, especially in moderate and severe BPD cases, demonstrates the importance to monitor vascular complications associated with BPD, that - in light with the co-occurrence of ROP - requires strategies to detect their intra- and extrapulmonary manifestation. PH - in preterm infants classified in group 3 of the Nice classification [43, 44] - is a serious consequence of elevated pulmonary vascular resistance in BPD resulting in the severe reduction of pulmonary blood flow and a subsequent decrease in oxygenation capacity [15, 45]. PH is observed in a quarter of infants with moderate to severe BPD [15, 16], but as well showed an association with immaturity per se [16], in line with our findings demonstrating that its occurrence is not fully explained by lung disease alone. The association of BPD with cardiac health is furthermore supported by the overall correlation of BPD with the presence of mild cardiac defects in NEuroSIS [39, 46], likely reflecting the increase in right ventricular afterload in BPD. The capacity of BPD severity (analysis of discriminatory power among morbidity occurrence) to capture some differences in morbidity risk support the results with an approximately tenfold higher risk of severe ROP in severe BPD cases (both cohorts) together with a 60% increase in risk for mild cardiac defects (NEuroSIS) and a tenfold increase in risk for PH (AIRR).

The importance of considering severity grading when characterizing risk profiles is underscored by, e.g., the differences obtained when comparing no BPD and moderate/severe BPD cases (analysis of discriminatory power among morbidity occurrence). At the same time, the lack of differences between no and mild BPD cases point to the need for better characterization of the clinically heterogeneous mild BPD group.

Despite the published risk of infants with BPD for adverse neurologic outcome [18, 47–49], BPD showed no relevant correlation with the presence of brain pathology. These findings have to be interpreted in the context of the (controversial) discussion about the co-occurrence of BPD and IVH or WMI [21, 39], diagnosed in about 50% of immature preterm infants [19], and its severe form cystic periventricular leukomalacia in 1.3% of preterm infants [50]. While risk factors such as oxygen supplementation and mechanical ventilation are shared between perinatal lung and brain pathology [17, 39], (lung mediated) inflammatory changes can add to the vulnerability of the fragile germinal matrix vasculature [51]. Studies agreed, however, that BPD is associated with delayed brain maturation [49]. Further, disturbances of central nervous system development might lay beyond the current detection limit for structural brain damage but could potentially be explained by e.g., network studies in the future.

Although suggested by previous studies [12, 14, 20, 40], correlations between (cardio)vascular complications such as PH, mild cardiac defects or ROP with perinatal brain pathology were not observed in either cohort.

The fact that none of the morbidities investigated had a significantly increased risk in all BPD severity groups in both cohorts, supported the limited overall discriminatory power of BPD as defined by our analysis of relative risk.

The simultaneous analysis of all morbidities revealed a broad spectrum of variations in morbidity profiles, spanning from patients with no morbidities to varying morbidity co-occurrences. Interestingly, around 20-25% of the patients in the cohorts exhibited isolated morbidities. This might be related to subclinical abnormalities that are not detected by current diagnostic processes but co-occurring with these *single* morbidities or the independence of morbidities in milder cases. The only exception from this observation was PH, which was consistently accompanied by other morbidities and never manifested alone, potentially indicating the severity of a vascular phenotype or its occurrence in severely diseased patients only.

### Identification of clusters of morbidity profiles

Using hierarchical clustering, we identified six patient clusters in AIRR and eight in NEuroSIS, based on similarity in morbidity profiles. While the varying morbidity distributions across clusters (Additional file 1, Supplementary Fig. 4) captures the complexity of morbidity profiles per se, we also identified the shared presence or absence of certain morbidities. This included the shared presence of moderate to severe BPD in cluster 4 or the presence of mild cardiac defects in clusters 5-8 as demonstrated in both cohorts. Likewise, the presence of perinatal brain pathology was observed in clusters 3, 4, 6 and 7 in the NEuroSIS cohort, whereas the presence of PH was specific to clusters 3 and 6 in AIRR and cluster 8 in NEuroSIS. In general, ROP was present in varying prevalences among all clusters. Our findings might thereby point to underlying (pathophysiologic) endotypes across organs. Whereas individual morbidities can only partially characterize specific clusters, e.g., cardiovascular complications are shared in clusters with higher morbidity load.

### Limitations

Despite the careful and comprehensive approach to delineate morbidity co-occurrences and profiles, future studies would benefit from addressing limitations of our study. Most importantly, differences in age distribution resulting from the cohorts’ inclusion criteria (i.e., < 32 weeks GA for AIRR; < 28 weeks GA for NEuroSIS) likely impact morbidity incidences and can explain the occurrence of more severe morbidity profiles as well as the higher death rate in NEuroSIS. Further, as our study analyzed morbidity profiles at discharge, the necessity to exclude infants who died during the NICU stay could introduce bias by excluding most severe morbidities. Next, variations between cohorts included differences in morbidity phenotyping of BPD (lung MRI, AIRR) and brain pathology (MRI of the central nervous system, AIRR). Limitations in the interpretation of the PH-associated results are caused by the rare incidence (AIRR: 15 cases, NEuroSIS: 4 cases), likely related to the limited capacity of echocardiography for detection of early stage PH [52]. Furthermore, clustering results are strongly impacted by the weight assigned to each morbidity. By using normalized ranks prior to clustering to scale morbidities to the same range of values, we assigned equal importance to all morbidities. However, attributing the same level of importance to the highest degree of BPD, perinatal brain pathology, or ROP severity and the presence of PH or mild cardiac defects might - despite a common design choice when performing clustering - oversimplify the complexity of their clinical relevance.

Despite these limitations, we demonstrated generalizability of our findings between cohorts, especially with respect to the correlation analysis and the discriminatory power of BPD.

## Conclusions

We broadly assessed the interrelated occurrence of morbidities at near-term age and identified not only individual associations, but also morbidity profiles based on *morbidity load*. While BPD showed significant pairwise correlations with ROP, PH, and mild cardiac defects, we demonstrated that morbidities are not always co-occurring when analyzing entire profiles or can even manifest isolated. Further, our analysis highlighted the limited power of BPD being a strong indicator of neurosensory and cardiovascular morbidity development until discharge, especially when considering mild BPD. Deep structural and functional phenotyping of the lung by imaging did not improve the correlation with discharge morbidities and, thus the characterization of risk profiles. Clustering patients based on similarities in morbidity profiles showed the complexity of morbidity co-occurrence and suggested that individual morbidities can only partially characterize clusters. At the same time, the occurrence of (cardio)vascular complications seem to be an important indicator of co-morbidity presence, potentially informing risk stratification and follow-up strategies in the future.

In summary, the clinical idea of a high risk profile, that is often conveyed to the parents during NICU stay or at discharge, needs careful revision as certain connections cannot be uniformly reproduced (e.g., an increased risk for perinatal brain pathology in any BPD group), while others surface (e.g., clustering of patients according to morbidity load associated with presence of PH, mild cardiac defects, or BPD grade). Further studies are needed to broaden our picture as well as re-analysis, when new diagnostic strategies or pathophysiological concepts emerge.

### Supplementary Information

**Additional file 1:** Supplementary material 1, Supplementary material 2, Supplementary Table 1, Supplementary Table 2, Supplementary Table 3, Supplementary Fig. 1, Supplementary Fig. 2, Supplementary Fig. 3, Supplementary Fig. 4, Supplementary Fig. 5.

## Data Availability

Datasets used and analyzed for the current study are available from the corresponding author on reasonable request.

## Abbreviations

ANCS: Antenatal corticosteroids
ASD: Atrial septal defect
BPD: Bronchopulmonary dysplasia
BW: Birth weight
CI: Confidence interval
EOI: Early onset infection
FiO_2_: Fraction of inspired oxygen
GA: Gestational age
IVH: Intraventricular hemorrhage
MRI: Magnetic resonance imaging
NFV: Net forward volume
OR: Odds ratio
PA: Pulmonary artery
PDA: Patent ductus arteriosus
PH: Pulmonary hypertension
PMA: Postmenstrual age
ROP: Retinopathy of prematurity
RR: Relative risk
WMI: White matter injury of prematurity
