# Supplementary material for: Delineating morbidity patterns in preterm infants at near-term age using a data-driven approach
Source: BMC Pediatr. 2024 Apr 11;24:249. doi: 10.1186/s12887-024-04702-5 (PMC11010410; doi:10.1186/s12887-024-04702-5)
Supplement: Supplementary file 1 — Additional file 1: Supplementary material 1, Supplementary material 2, Supplementary Table 1, Supplementary Table 2, Supplementary Table 3, Supplementary Fig. 1, Supplementary Fig. 2, Supplementary Fig. 3, Supplementary Fig. 4, Supplementary Fig. 5. [file 12887_2024_4702_MOESM1_ESM.pdf]

Additional file 1. Collection of clinical data and additional results

- Supplementary material 1. Collection of clinical data in the AIRR cohort
- Supplementary material 2. Collection of clinical data in the NEuroSIS cohort
- Supplementary table 1. Overview of selected variables and their statistics in AIRR and NEuroSIS cohorts.
- Supplementary table 2. Morbidity prevalences and ORs stratified by BPD severity groups.
- Supplementary table 3. Correlation matrix of morbidities in AIRR cohort including (a) structural and (b) functional lung variables.
- Supplementary fig. 1. Morbidity pattern distribution in (a) AIRR and (b) NEuroSIS.
- Supplementary fig. 2. Silhouette coefficient for different numbers of clusters k in (a) AIRR and (b) NEuroSIS.
- Supplementary fig. 3. Cluster sizes in (a) AIRR and (b) NEuroSIS.
- Supplementary fig. 4. Prevalence of morbidities among clusters in (a) AIRR and (b) NEuroSIS.
- Supplementary fig. 5. Distribution of perinatal variables among clusters in (a) AIRR and (b) NEuroSIS.

Supplementary material 1. Collection of clinical data in the AIRR cohort

During pregnancy, several risk factors for fetal development (i.e., gestational diabetes, HELLP syndrome [1], the history of infection), intrauterine growth restriction (birth weight (BW) $<$ 10th percentile) as well as prenatal maternal glucocorticoid administration (given antenatal corticosteroids (ANCS) 7 days to 24 h before birth [2]) were recorded. Perinatal variables included the type of delivery, performance of tocolysis, premature rupture of the membranes more than 12 hours prenatally and signs of amniotic infection syndrome, among others. The baseline information at birth included biometric data (e.g., postmenstrual age (PMA), sex, birth weight and body size) and postnatal adaptation (i.e., arterial pH of umbilical cord, Apgar score after 1 and 5 min, clinical risk index for babies (CRIB score) [3]).

Postnatal monitoring included variables describing the pulmonary, cardiovascular and neurologic development of the infant. Lung health was characterized by the severity of respiratory distress syndrome (RDS) [4], the need for postnatal corticosteroids or surfactant [5–7], type and duration of ventilation support (days of intermittent positive pressure ventilation, nasal intermittent positive pressure ventilation or continuous positive airway pressure ventilation; one day counts as more than 4 hours of ventilation) as well as hyperoxia exposure (one day counts as more than 12 hours of oxygen supply, fraction of inspired oxygen (FiO_2) $>$ 0.21). X-Ray was used to detect the presence of pneumothorax or pulmonary emphysema [8]. Pulmonary hypertension (PH) was diagnosed at 36 weeks PMA by echocardiography finding of tricuspid regurgitation and/or moderate or severe dilatation of the right atrium/ventricle [9–11]. The cardiovascular course was further described by the need of treatment for patent ductus arteriosus (PDA), results of routine heart echocardiography examinations comprising function of left and right heart, pulmonary vessel pressures, intracardiac and extracardiac shunts (e.g., septal defects), or other congenital heart malformations. Systemic infections (e.g., early onset infection (EOI)) were diagnosed according to Sherman et al. [12] with one or more clinical and laboratory signs of infection. They were differentiated in primary and secondary pneumonia or sepsis.

Supplementary material 2. Collection of clinical data in the NEuroSIS cohort

In the NEuroSIS trial, information about mother (e.g. age, race, use of ANCS, mode of birth, infection) as well as the infant was recorded, as published previously [13]. Biometric data (e.g., birth weight, GA, sex), postnatal adaptation (e.g., APGAR score) and respiratory disease (e.g., days of oxygen supplementation, ventilation mode) were collected. Comorbidity development was assessed including the diagnosis of retinopathy of prematurity (ROP) and medically treated PDA according to international criteria. For further information refer to Bassler et al. [13].

Supplementary table 1 Overview of selected variables and their statistics in AIRR and NEuroSIS cohorts.

	AIRR		NEuroSIS	
	n	Range	n	Range
GA [weeks]	171	23.14-31.57	359	23.14-27.86
BW [g]	171	300-1770	359	340-1300
Body length [cm]	171	25.0-43.0	-	-
Sex	171	{female, male}	359	{female, male}
Multiple birth	171	{0, 1, 2}	359	{0, 1, 2, 3}
CRIB score	171	0-16	-	-
Umbilical artery pH	160	6.95-7.49	231	6.84-7.64
1-minute Apgar	171	1-9	351	0-9
5-minute Apgar	171	4-10	333	1-9
ANCS	165	{0, 1, 2}	359	{0, 1}
Hospital stay [d]	156	16-152	358	51-369
EOI	167	{no, yes}	359	{no, yes}
RDS	165	0.0-4.0	-	-
BPD	171	{0, 1, 2, 3}	359	{0, 1, 2, 3}
PH	171	{no, yes}	359	{no, yes}
Mild cardiac defect	171	{no, yes}	359	{no, yes}
Perinatal brain pathology	171	{no, yes}	359	{no, yes}
ROP	171	{0, 1, 2, 3, 4}	359	{0, 1, 2, 3, 4}
Struct. lung abnormality	70	1.0-4.0	-	-
PA flow	85	-3.77-1.30	-	-

Per variable, we list the number of patients for which the variable has been recorded and the range of possible values.

Abbreviations: GA = gestational age; BW = birth weight; CRIB score = clinical risk index for babies; ANCS = antenatal corticosteroids; EOI = early onset infection ţtepSherman1980-hb; RDS = respiratory distress syndrome; BPD = bronchopulmonary dysplasia [14]; PH = pulmonary hypertension [15, 16]; ROP = retinopathy of prematurity [17]; PA flow = pulmonary artery flow.

Supplementary table 2 Morbidity prevalences and ORs stratified by BPD severity groups.

(a) AIRR

	**No BPD** n = 59	**Mild BPD** n = 58	**Moderate BPD** n = 24	**Severe BPD** n = 30
**PH**				
Count	1 (1.69%)	4 (6.9%)	5 (20.83%)	5 (16.67%)
OR	-	4.25 (0.40-215.15)	14.69 (1.51-733.85)	11.27 (1.18-557.79)
**Mild cardiac defect**				
Count	16 (27.12%)	17 (29.31%)	11 (45.83%)	14 (46.67%)
OR	-	1.11 (0.46-2.70)	2.25 (0.75-6.79)	2.33 (0.85-6.49)
**Perinatal brain pathology (mild)**				
Count	10 (16.95%)	18 (31.03%)	7 (29.17%)	9 (30.0%)
OR	-	2.19 (0.85-5.95)	2.0 (0.55-6.95)	2.08 (0.65-6.67)
**Perinatal brain pathology (severe)**				
Count	8 (13.56%)	8 (13.79%)	1 (4.17%)	3 (10.0%)
OR	-	1.02 (0.31-3.39)	0.28 (0.01-2.30)	0.71 (0.11-3.28)
**ROP (grades 1/2)**				
Count	7 (11.86%)	10 (17.24%)	8 (33.33%)	13 (43.33%)
OR	-	1.54 (0.48-5.18)	3.65 (0.99-13.93)	5.55 (1.73-19.39)
**ROP (grades $\geq$ 3)**				
Count	1 (1.69%)	4 (6.9%)	1 (4.17%)	5 (16.67%)
OR	-	4.25 (0.40-215.15)	2.49 (0.03-201.29)	11.27 (1.18-557.79)

(b) NEuroSIS

	**No BPD** n = 115	**Mild BPD** n = 94	**Moderate BPD** n = 39	**Severe BPD** n = 111
**PH**				
Count	0 (0.0%)	1 (1.06%)	0 (0.0%)	3 (2.7%)
OR	-	- (-)	- (-)	- (-)
**Mild cardiac defect**				
Count	46 (40.0%)	48 (51.06%)	25 (64.1%)	71 (63.96%)
OR	-	1.56 (0.87-2.82)	2.66 (1.19-6.17)	2.65 (1.50-4.73)
**Perinatal brain pathology (mild)**				
Count	50 (43.48%)	42 (44.68%)	18 (46.15%)	60 (54.05%)
OR	-	1.05 (0.58-1.89)	1.11 (0.50-2.46)	1.53 (0.88-2.67)
**Perinatal brain pathology (severe)**				
Count	10 (8.7%)	10 (10.64%)	6 (15.38%)	9 (8.11%)
OR	-	1.25 (0.44-3.52)	1.9 (0.53-6.30)	0.93 (0.32-2.66)
**ROP (grades 1/2)**				
Count	38 (33.04%)	38 (40.43%)	16 (41.03%)	35 (31.53%)
OR	-	1.37 (0.75-2.52)	1.41 (0.62-3.16)	0.93 (0.51-1.69)
**ROP (grades $\geq$ 3)**				
Count	2 (1.74%)	11 (11.7%)	8 (20.51%)	25 (22.52%)
OR	-	7.42 (1.56-70.70)	14.26 (2.66-144.50)	16.27 (3.88-145.47)

Displayed per each BPD severity group are: morbidity count with the corresponding incidence relative to the size of the respective BPD severity group and the odds ratio (OR) with the corresponding 95% confidence interval (CI).

Supplementary table 3 Correlation matrix of morbidities in AIRR cohort including (a) structural and (b) functional lung variables.

(a) On the n=70 patients with available data on structural lung abnormality.

	1	2	3	4	5	6
1 BPD	-	0.292	0.106	0.033	0.293	0.343*
2 PH	0.292	-	0.022	0.043	0.133	0.040
3 Mild cardiac defect	0.106	0.022	-	-0.083	-0.037	0.153
4 Perinatal brain pathology	0.033	0.043	-0.083	-	-0.140	0.124
5 ROP	0.293	0.133	-0.037	-0.140	-	0.182
6 Struct. lung abnormality	0.343*	0.040	0.153	0.124	0.182	-

(b) On the n=85 patients with available data on PA flow.

	1	2	3	4	5	6
1 BPD	-	0.305*	0.079	0.032	0.261	-0.178
2 PH	0.305*	-	0.114	-0.015	0.123	0.014
3 Mild cardiac defect	0.079	0.114	-	-0.080	0.019	-0.197
4 Perinatal brain pathology	0.032	-0.015	-0.080	-	-0.046	-0.054
5 ROP	0.261	0.123	0.019	-0.046	-	-0.179
6 PA flow	-0.178	0.014	-0.197	-0.054	-0.179	-

Entries represent Kendall's τ coefficient measuring strength of correlation. The morbidities BPD, perinatal brain pathology, and ROP are ordinal, PH and mild cardiac defects are binary, whereas PA flow and structural lung abnormalities are continuous.

*Statistically significant correlation after Bonferroni correction (adjusted p-value $<$ 0.05).

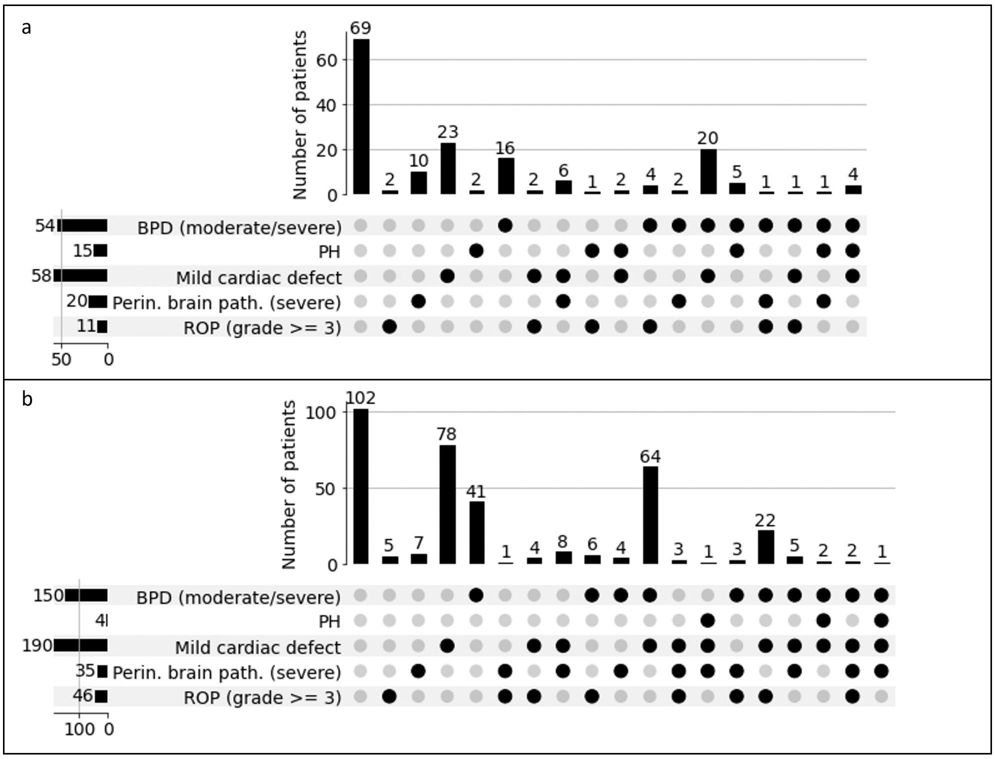

Supplementary fig. 1 Morbidity pattern distribution in (a) AIRR and (b) NEuroSIS. Each column corresponds to a different morbidity pattern, with the black dots indicating the morbidities present in each pattern and the vertical bar showing the number of patients exhibiting the respective pattern. The horizontal bars on the left indicate the total number of patients exhibiting each morbidity. For visualization purposes, we used a binary representation of morbidities, displaying a black dot for moderate and severe BPD, severe perinatal brain pathology, and ROP grades ≥ 3.

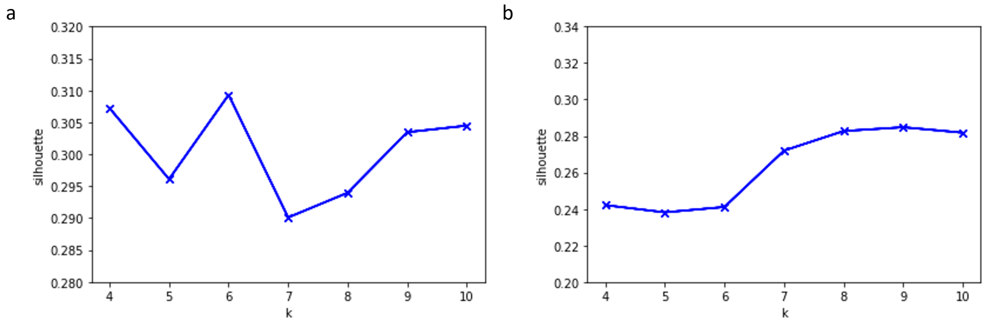

Supplementary fig. 2 Silhouette coefficient for different numbers of clusters k in (a) AIRR and (b) NEuroSIS.

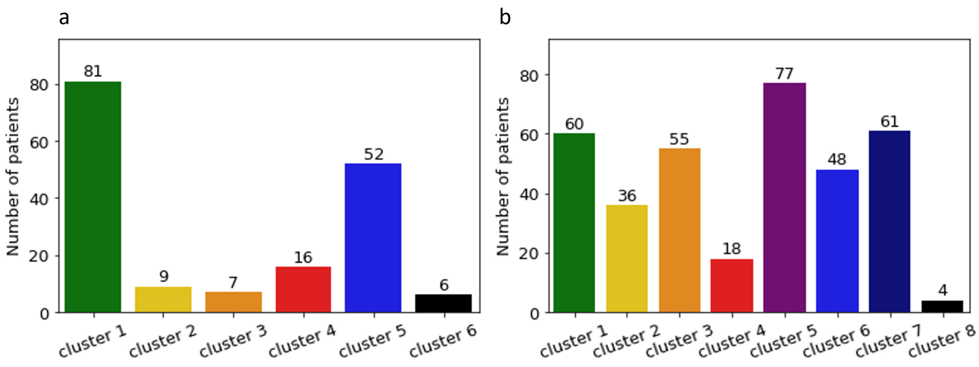

Supplementary fig. 3 Cluster sizes in (a) AIRR and (b) NEuroSIS.
The bars indicate the number of patients assigned to each cluster based on their morbidity profiles.

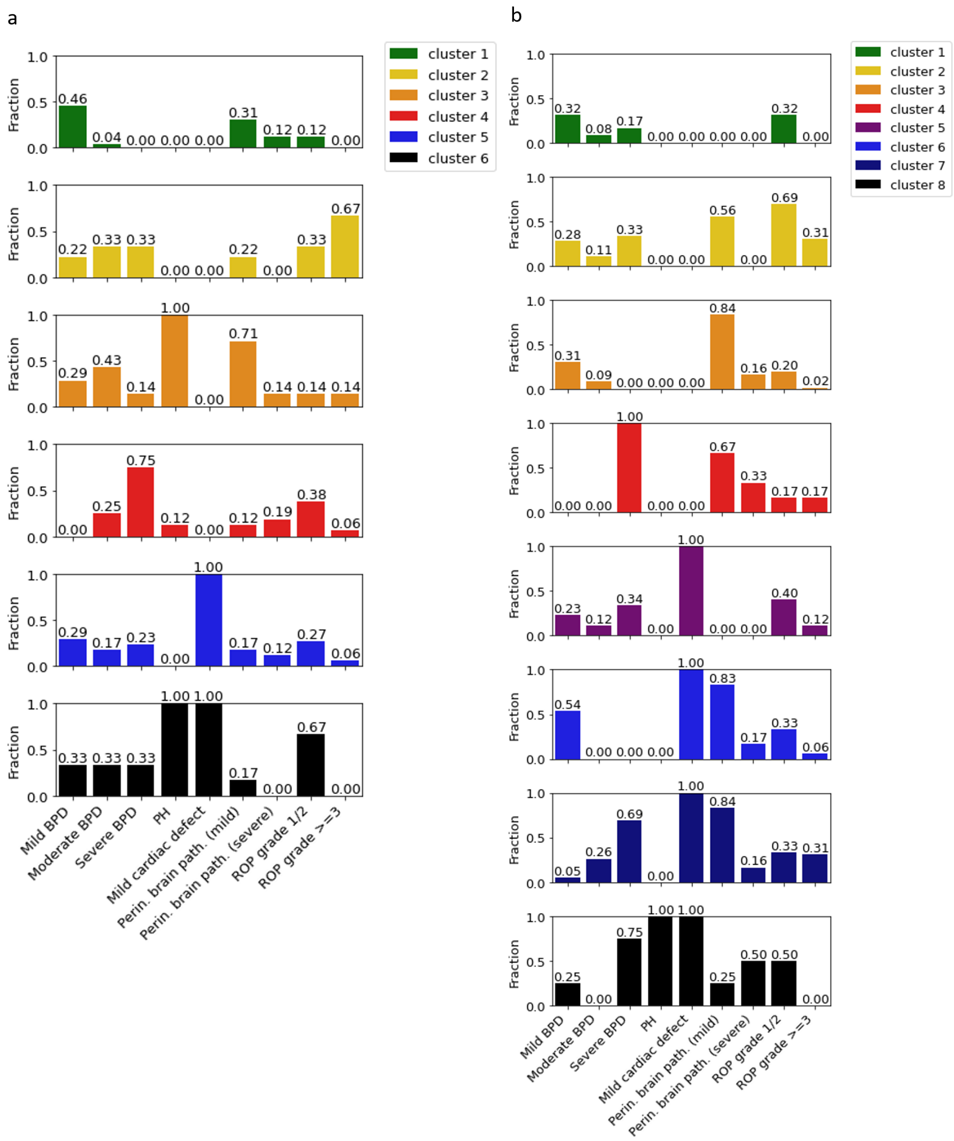

Supplementary fig. 4 Prevalence of morbidities among clusters in (a) AIRR and (b) NEuroSIS.

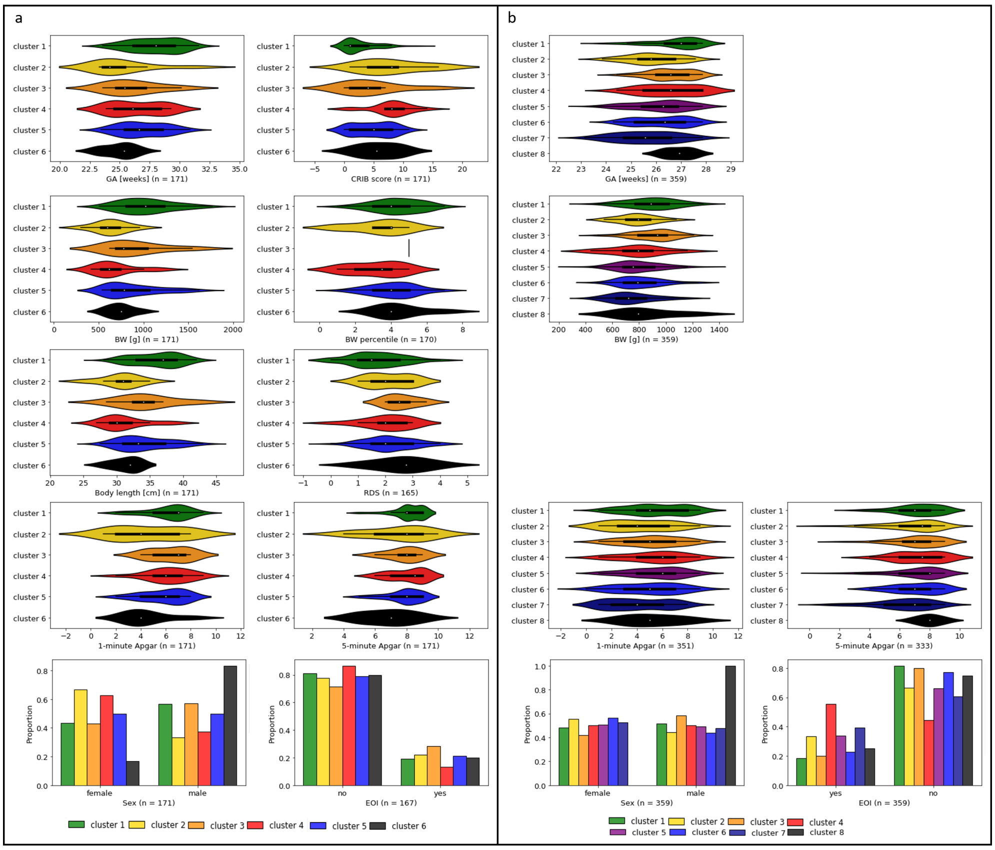

Supplementary fig. 5 Distribution of perinatal variables among clusters in (a) AIRR and (b) NEuroSIS.
GA = gestational age, CRIB = clinical risk index for babies, BW = birth weight, RDS = respiratory distress syndrome, EOI = early onset infection. The BW percentile is encoded as follows: 1 for percentiles below 3; 2 for percentiles 3-10; 3 for percentiles 10-25; 4 for percentiles 25-50; 5 for percentiles 50-75; 6 for percentiles 75-90; 7 as percentiles 90-97.

References

[1] Weinstein L. Syndrome of hemolysis, elevated liver enzymes, and low platelet count: a severe consequence of hypertension in pregnancy. Am J Obstet Gynecol. 1982;142(2):159–67.

[2] Crowther CA, Haslam RR, Hiller JE, Doyle LW, Robinson JS. Neonatal respiratory distress syndrome after repeat exposure to antenatal corticosteroids: a randomised controlled trial. Lancet. 2006;367(9526):1913–9.

[3] Network TIN. The CRIB (clinical risk index for babies) score: a tool for assessing initial neonatal risk and comparing performance of neonatal intensive care units. Lancet. 1993;342(8865):193–8.

[4] Couchard M, Polge J, Bomsel F. Hyaline membrane disease: diagnosis, radiologic surveillance, treatment and complications. Ann Radiol. 1974;17(7):669–83.

[5] Speer CP. Neonatales Atemnotsyndrom. In: Reinhardt D, editor. Therapie der Krankheiten im Kindes-und Jugendalter. 7th ed. Berlin (DE): Springer; 2004. p. 14–19.

[6] Jobe AH, Ikegami M. Antenatal infection/inflammation and postnatal lung maturation and injury. Respir Res. 2001;2:1–6.

[7] Kari MA, Eronen M, Virtanen M, Hallman M, Teramo K, Koivisto M, et al. Prenatal dexamethasone treatment in conjunction with rescue therapy of human surfactant: a randomized placebo-controlled multicenter study. Pediatrics. 1994;93(5):730–6.

[8] Swischuk LE. Bubbles in hyaline membrane disease: differentiation of three types. Radiology. 1977;122(2):417–26.

[9] Mourani PM, Sontag MK, Younoszai A, Miller JI, Kinsella JP, Baker CD, et al. Early pulmonary vascular disease in preterm infants at risk for bronchopulmonary dysplasia. Am J Respir Crit Care Med. 2015;191(1):87–95.

[10] Arjaans S, Zwart EAH, Ploegstra MJ, Bos AF, Kooi EMW, Hillege HL, et al. Identification of gaps in the current knowledge on pulmonary hypertension in extremely preterm infants: A systematic review and meta-analysis. Paediatr Perinat Epidemiol. 2018;32(3):258–67.

[11] Simonneau G, Montani D, Celermajer DS, Denton CP, Gatzoulis MA, Krowka M, et al. Haemodynamic definitions and updated clinical classification of pulmonary hypertension. Eur Respir J. 2019;53(1):1801913.

[12] Sherman MP, Goetzman BW, Ahlfors CE, Wennberg RP. Tracheal asiration and its clinical correlates in the diagnosis of congenital pneumonia. Pediatrics.

1980;65(2):258–63.

[13] Bassler D, Plavka R, Shinwell ES, Hallman M, Jarreau PH, Carnielli V, et al. Early inhaled budesonide for the prevention of bronchopulmonary dysplasia. N Engl J Med. 2015;373(16):1497–506.

[14] Jobe AH, Bancalari E. Bronchopulmonary dysplasia. Am J Respir Crit Care Med. 2001;163(7):1723–9.

[15] Apitz C, Hansmann G, Schranz D. Hemodynamic assessment and acute pulmonary vasoreactivity testing in the evaluation of children with pulmonary vascular disease. Expert consensus statement on the diagnosis and treatment of paediatric pulmonary hypertension. The European Paediatric Pulmonary Vascular Disease Network, endorsed by ISHLT and DGPK. Heart. 2016;102 Suppl 2:ii23–9.

[16] Hansmann G, Sallmon H, Roehr CC, Kourembanas S, Austin ED, Koestenberger M, et al. Pulmonary hypertension in bronchopulmonary dysplasia. Pediatr Res. 2021;89(3):446–55.

[17] Chiang MF, Quinn GE, Fielder AR, Ostmo SR, Paul Chan RV, Berrocal A, et al. International Classification of Retinopathy of Prematurity, Third Edition. Ophthalmology. 2021;128(10):e51–e68.
